# A Comparative Quantitative LC-MS/MS Profiling Analysis of Human Pancreatic Adenocarcinoma, Adjacent-Normal Tissue, and Patient-Derived Tumour Xenografts

**DOI:** 10.3390/proteomes6040045

**Published:** 2018-11-06

**Authors:** Orla Coleman, Michael Henry, Fiona O’Neill, Sandra Roche, Niall Swan, Lorraine Boyle, Jean Murphy, Justine Meiller, Neil T. Conlon, Justin Geoghegan, Kevin C. Conlon, Vincent Lynch, Ninfa L. Straubinger, Robert M. Straubinger, Gerard McVey, Michael Moriarty, Paula Meleady, Martin Clynes

**Affiliations:** 1 National Institute for Cellular Biotechnology, Dublin City University, Glasnevin, Dublin 9, Ireland; michael.henry@dcu.ie (M.H.); fiona.oneill@dcu.ie (F.O.); sandra.roche@dcu.ie (S.R.); justine.meiller@dcu.ie (J.M.); neil.conlon4@mail.dcu.ie (N.T.C.); glynch96@gmail.com (V.L.); michaelmoriartyirl@gmail.com (M.M.); paula.meleady@dcu.ie (P.M.); martin.clynes@dcu.ie (M.C.); 2 St. Vincent’s University Hospital, Dublin 4, Ireland; nswan@svhg.ie (N.S.); L.Boyle@st-vincents.ie (L.B.); J.Murphy@st-vincents.ie (J.M.); geoghegj@indigo.ie (J.G.); conlonk@tcd.ie (K.C.C.); gerard.mcvey@slh.ie (G.M.); 3 Trinity College Dublin, College Green, Dublin 2, Ireland; 4 Department of Pharmaceutical Sciences, University at Buffalo, State University of New York, Buffalo, NY 14214, USA; Nls2@buffalo.edu (N.L.S.); Rms@buffalo.edu (R.M.S.); 5 St. Luke’s Hospital, Highfield Road, Rathgar, Dublin 6, Ireland

**Keywords:** pancreatic cancer, proteomics, ADC therapy, PDX, membrane-enriched

## Abstract

Pancreatic ductal adenocarcinoma (PDAC) is one of the deadliest cancers worldwide; it develops in a relatively symptom-free manner, leading to rapid disease progression and metastasis, leading to a 5-year survival rate of less than 5%. A lack of dependable diagnostic markers and rapid development of resistance to conventional therapies are among the problems associated with management of the disease. A better understanding of pancreatic tumour biology and discovery of new potential therapeutic targets are important goals in pancreatic cancer research. This study describes the comparative quantitative LC-MS/MS proteomic analysis of the membrane-enriched proteome of 10 human pancreatic ductal adenocarcinomas, 9 matched adjacent-normal pancreas and patient-derived xenografts (PDXs) in mice (10 at F1 generation and 10 F2). Quantitative label-free LC-MS/MS data analysis identified 129 proteins upregulated, and 109 downregulated, in PDAC, compared to adjacent-normal tissue. In this study, analysing peptide MS/MS data from the xenografts, great care was taken to distinguish species-specific peptides definitively derived from human sequences, or from mice, which could not be distinguished. The human-only peptides from the PDXs are of particular value, since only human tumour cells survive, and stromal cells are replaced during engraftment in the mouse; this list is, therefore, enriched in tumour-associated proteins, some of which might be potential therapeutic or diagnostic targets. Using human-specific sequences, 32 proteins were found to be upregulated, and 113 downregulated in PDX F1 tumours, compared to primary PDAC. Differential expression of CD55 between PDAC and normal pancreas, and expression across PDX generations, was confirmed by Western blotting. These data indicate the value of using PDX models in PDAC research. This study is the first comparative proteomic analysis of PDAC which employs PDX models to identify patient tumour cell-associated proteins, in an effort to find robust targets for therapeutic treatment of PDAC.

## 1. Introduction

Pancreatic ductal adenocarcinoma (PDAC), which constitutes 90% of pancreatic cancers, is one of the most lethal solid malignancies, and the fourth leading cause of cancer-related mortality in the world. Despite the improvement in screening and therapies of many solid tumours, pancreatic cancer prognosis remains dismal with 91% of patients not surviving within five years of diagnosis, and only 26% of patients surviving within the first year of diagnosis (www.pancreatic.org). The absence of symptoms in its initial stages, and insufficient early detection tools, lead to rapid disease progression, distant metastases, and poor prognoses; it is estimated that only 10% of presented patients have tumours that are potentially curable by resection [1,2] (www.ncri.ie). For most of the cases, this cancer is advanced, unresectable, and metastatic, and for the minority of patients who are diagnosed at an early stage, conventional cancer treatments have limited benefit and little impact on disease progression. 

Since 1997, gemcitabine therapy has been the standard first-line treatment for patients with unresectable locally advanced or metastatic pancreatic cancer [3]. Most recently, a phase III trial of adjuvant modified FOLFIRINOX significantly improved disease-free survival, metastatis free survival and overall survival compared to gemcitabine, among patients with surgically removed PDAC, and is now being considered a new standard of care for pancreatic cancer after resection [4]. Monoclonal antibodies have proved to have a successful role in cancer therapy, specifically antibody-drug conjugate (ADC) therapy is emerging as a chemotherapeutic with great promise for solid cancer treatment [5]. With the introduction of such targeted therapeutics onto the market, pancreatic cancer research is focused on the identification of novel drug targets to produce immunoconjugates against this disease. Choosing the appropriate target for ADCs is a critical step that affects the efficacy of these biotherapeutics [6]. 

Besides the lack of effective treatments for pancreatic cancer at present, serum carbohydrate 19-9 (CA19-9) is the only pancreatic cancer biomarker (prognostic biomarker only) approved for use by the US Food and Drug Administration (FDA). CA19-9 can be elevated in many types of gastrointestinal cancers, such as colorectal cancer and oesophageal cancer, as well as in patients with pancreatitis, obstructive jaundice, cholangitis, cirrhosis, and diseases of the bile ducts [7,8]. Unfortunately, despite sensitivity and specificity of approximately 79% and 82%, respectively, CA-19-9 is inadequate for the early detection of PDAC in asymptomatic patients, and cannot satisfactorily diagnose PDAC; however, if elevated, it is useful in following patients with known disease. The lack of diagnostic biomarkers and general screening results in an inability to detect early-stage pancreatic cancer, which enables metastasis to occur, and significantly decreases survival rates. This situation emphasises the need for more specific biomarkers against pancreatic cancer to be identified, so that it can be diagnosed at an early stage to prevent poor outcomes for patients. 

Comprehensive reviews by Coleman et al. [9] and Pan et al. [10], among others, outlines proteomic studies in pancreatic cancer, to date. Although there have been reports of potential biomarkers and diagnostic targets, none have been clinically approved. Many proteomic profiling studies have been performed on cell lines [11,12,13], but great care must be taken when studying cell lines as they are cultured repeatedly for years, and may not retain the cancer proteome of the original patient. Pancreatic tissue proteomics has made some significant findings, largely due to the increase in sensitivity of mass spectrometers, and an improvement in the associated preparations. Isotope-coded affinity tag technology (ICAT) has been used to identify proteins in PDAC tissues which are involved in invasion and metastasis [14]. A similar study using ICAT found that 50% of proteins overexpressed in pancreatitis were previously identified in pancreatic cancer studies, and these proteins potentially contribute to false-positive biomarkers of pancreatic cancer [15]. Multiple studies have identified galectin-1 as overexpressed in PDAC tissues through various proteomic approaches, including 2-dimensional gel electrophoresis, Western blotting, immunohistochemistry, and mass spectrometry [16,17]. More recently, it has been established as a stromal protein in PDAC, which mediates tumour–stroma crosstalk to regulate PDAC progression, and suggests it has therapeutic potential [18]. Membrane proteomic studies of PDAC, to date, are limited to cell line analyses [19,20]. Cell surface glycoprotein labelling or membrane enrichment kits, followed by mass spectrometry, were the methods of choice for membrane proteome profiling in these studies. 

This study set out to compare the proteomes of a panel of human pancreatic adenocarcinomas, adjacent-normal tissue, and patient-derived xenografts (PDX), from these tumours. The PDX sample set were analysed at the first and second generation in mice—F1 and F2. The tumours selected were characterised by a consultant histopathologist. The xenograft samples were analysed to determine their stability as models for PDAC, and maintaining the PDAC proteome across generations of PDX mice. We specifically enriched membrane and membrane-associated proteins from the samples as candidate membrane proteins found to be overexpressed in the pancreatic tumours (primary and/or xenograft) have potential as targets for novel targeted therapeutics, such as antibody–drug conjugates or monoclonal antibody therapies. Through the engraftment process, and as the tumours grow in mice, the patient stroma is overtaken with tissue of mouse origin [21]. This allows an interrogation of the complex tumour/stroma relationship. On the proteomic level, using high-resolution mass spectrometry, we have selectively determined the tumour cell derived human-specific proteins contributing to PDAC tumorigenesis. This study is an important step forward for PDAC research, and presents tumour-associated proteins which have potential as biomarkers or targets for ADC therapy or other antibody-derived targeted therapies. This study presents the first membrane protein analysis of PDAC primary tissues and PDX PDAC tissues. 

## 2. Materials and Methods 

### 2.1. Patient Demographics and PDX Information

Ten pancreatic cancer primary tumour tissue specimens and 9 normal-adjacent specimens, which were patient-matched, were obtained from St. Vincent’s University Hospital (SVUH), Dublin, between 2013 and 2015. All patients were undergoing pancreatic adenocarcinoma resection. The average age of the patients was 66 years old, of those, *n* = 7 are male and *n* = 4 are female. All samples were collected and processed in compliance with the SVUH and Dublin City University (DCU) ethics committees. Pancreatic adenocarcinoma patient-derived xenograft (PDX) tissues were generated by subcutaneous seeding in severe combined immunodeficiency disease (SCID) mice in-house at DCU. Twenty successful PDX tissues, *n* = 10 for both the F1 and F2 generations, were used for this study. F1 generation refers to mice subcutaneously engrafted with primary patient tumour material, whereas the F2 generation of mice were injected with a fragment of the F1 tumour. All samples were cryopreserved at −80 °C on the day of extraction, until sample preparation was performed. Representative tumour tissue was formalin-fixed and paraffin-embedded for each PDX tumour. Patient and sample details are provided in Supplementary Table S1. Primary patient samples were confirmed as pancreatic adenocarcinoma by a pathologist (N.S.) in SVUH. PDX samples were also confirmed by pathology examination to maintain the human tumour content and morphology of the original tumour.

### 2.2. Membrane Protein Enrichment and Protein Digestion

Tissue specimens were subjected to membrane protein enrichment using the Mem-PER Plus Membrane Protein Extraction Kit (Thermo Fisher Scientific, Waltham, MA, USA) which applies a mild detergent-based selective extraction protocol to enrich integral membrane proteins and membrane-associated proteins. The extraction was performed essentially according to manufacturer’s instructions for hard tissue, except that buffer volumes were adjusted for the samples depending on the weight. The membrane-enriched fraction was used for all further analysis of the samples in this study. The membrane-enriched fraction from each of the tissue samples was cleaned up using the ReadyPrep 2D Clean-Up Kit (Bio-Rad, CA, USA), according to manufacturer’s instructions. The cleaned protein pellets were resuspended in a buffer containing 6 M urea, 2 M thiourea, and 10 mM Tris, pH 8.5, and assayed for protein concentration using the QuickStart Bradford Protein Assay (Bio-Rad). Fifteen micrograms of protein were suspended with 50 mM ammonium bicarbonate, and protein digestion was carried out as previously described [22]. 

### 2.3. Quantitative Label-Free LC-MS/MS and Data Analysis

Nano LC-MS/MS analysis was carried out using a Dionex Ultimate 3000 RSLCnano system (Thermo Fisher Scientific) coupled to a hybrid linear ion trap/Orbitrap mass spectrometer (LTQ Orbitrap XL; Thermo Fisher Scientific). LC-MS/MS methods were applied as previously described [22]. Quantitative label-free data analysis was carried out using Progenesis QI for Proteomics (version 2.0; Nonlinear Dynamics, a Waters company, Newcastle upon Tyne, UK), essentially as recommended by the manufacturer (www.nonlinear.com). Peptide and protein identification were achieved with Proteome Discoverer 2.1 using Sequest HT (Thermo Fisher Scientific), Mascot, and Percolator. Data for the membrane-enriched fraction of all tumours and adjacent-normal differential analyses were searched against the NCBI UniProt Swiss-Prot *Homo sapiens* database containing 20,148 sequences (fasta file downloaded May 2017). Data for the PDX analyses were searched against a dual human/mouse fasta database to accurately determine the origin of the protein identifications, i.e., primary human tumour or SCID mice stroma. This database was created in-house by merging the NCBI UniProt Swiss-Prot *Homo sapiens* database containing 20,148 sequences (fasta file downloaded May 2017) and NCBI UniProt Swiss-Prot *Mus musculus* database containing 16,863 sequences (fasta file downloaded May 2017). 

To better analyse the PDX model and evaluate the tumour/stroma (human/mouse) origin of the orthologous proteins in the xenograft experiments, only peptide spectrum matches (PSMs) matching to (1) an unambiguous protein and (2) 1 protein identification, were allowed. The PSM option omits peptides that cannot be unambiguously matched to one protein, i.e., if a PSM matches two proteins, the mouse and human version of the same protein, it is omitted; therefore, only species-specific PSMs and, thus, peptides, are retained. If evidence for BOTH human and mouse peptides from an orthologous protein were observed, then peptides that cannot distinguish between the two species were ignored. Assembly of unambiguous, species-specific, confidently identified PSMs resulted in an approximate loss of two-thirds of the proteomic data. However, all differential peptide data carried forward for analysis with Progenesis QI could now be confidently distinguished from peptides derived from the tumour, and those orthologues with the mouse form of the protein.

The following search parameters were used for protein identification: (1) peptide mass tolerance set to 20 ppm, (2) MS/MS mass tolerance set to 0.6 Da, (3) up to two missed cleavages were allowed, (4) carbamidomethylation of cysteine set as a fixed modification, and (5) methionine oxidation set as a variable modification. Only highly confident peptide identifications with a false discovery rate (FDR) ≤ 0.01 (identified using a SEQUEST HT workflow coupled with Percolator validation in Proteome Discoverer 2.1 (Thermo Fisher Scientific)) were imported into Progenesis QI software for further analysis. Protein identifications were reviewed, and only those which passed the following criteria were considered differentially expressed between experimental groups with high confidence and statistical significance: (i) an ANOVA *p*-value of ≤0.05 between experimental groups; (ii) proteins with ≥2 unique peptides contributing to the identification; (iii) a ≥1.5-fold change in relative abundance between the two experimental conditions. For the patient-matched analysis of adjacent-normal tissues and primary PDAC, a repeated measures ANOVA was used for paired sample statistical analysis. To calculate the maximum fold change for a protein, Progenesis calculates the mean abundance for that protein in each experimental condition. These mean values are then placed in a condition-vs-condition matrix to find the maximum fold change between any two conditions’ mean protein abundances. The mass spectrometry proteomics data have been deposited to the ProteomeXchange Consortium via the PRIDE partner repository.

Data visualization was achieved using violin plots and heatmaps, which were generated using the ggplot2 package in R. We determined the coefficient of variation (CV) of the patients for each differentially expressed protein, and plotted the CV distribution as violin plots. Violin plots are similar to boxplots, but instead of showing summary statistics, such as median and interquartile ranges, the violin plot shows the full variable distribution of all the protein abundances, with the widest points of the violin indicating the most common CV values. Violin plots were generated using R-studio and the ggplot2 library. The relative expression levels of the differentially expressed proteins across the experimental groups was visualised using heatmaps generated with ggplot2. 

### 2.4. Immunohistochemistry

All immunohistochemical (IHC) staining was performed using the DAKO Autostainer (DAKO, Glostrup, Denmark). Antigen retrievals were performed using the Epitope Retrieval 3-in-1 solution (pH6) (DAKO). For epitope retrieval, slides were heated to 97 °C for 20 min, and then cooled to 65 °C. DAKO Real Envision Detection System, Peroxidase/DAB+ detection system was used.

On the autostainer (DAKO, Glostrup, Denmark), slides were blocked for 10 min with 200 µL horseradish peroxidase Block (DAKO). Slides were washed with 1× wash buffer, and 200 µL of the primary antibody was added to the slides for 30 min. Slides were once again washed with 1× wash buffer, and then incubated with 200 µL Real EnVision (DAKO) for 30 min. Slides were washed, again, with wash buffer, and then stained with 200 µL DAB+ substrate for 5 min, and this procedure was repeated twice. All slides were then counterstained with haematoxylin (DAKO) for 5 min, and were rinsed with deionised water, and then with wash buffer. A negative control (NC) sample was also tested for each sample using antibody diluent without any antibody present. This was used to evaluate any non-specific staining. Following the counterstaining with haematoxylin, the slides were then dehydrated. This was achieved by immersing the slide in 70%, 90%, and 100% EtOH, twice each in EtOH solution for 3 min. The slides were then immersed into xylene, twice, for 5 min each. Once the slides were cleared, they were mounted using distyrene, a plasticizer, and xylene (DPX) (BDH Laboratories, Poole, UK). In order to visualise and evaluate the amount of mouse stromal infiltration into the human tumour cells and tissue, a mouse monoclonal antibody to human mitochondria (1/1000 Ab92824, Abcam, Cambridge, UK) was used. 

### 2.5. Western Blotting

Equal amounts (10 µg) of protein corresponding to samples of the membrane-enriched fraction were prepared in Laemmli SDS-PAGE loading buffer, and denatured for 5 min at 95 °C. Samples were loaded onto 4–12% NuPAGE Bis-Tris gels, and separated at 180 V for 1 h. PageRuler Plus pre-stained protein ladder (Thermo Fisher Scientific) was used as the molecular weight marker. Electrophoretic transfer was achieved using a Power Blotter (Thermo Fisher Scientific) onto nitrocellulose membrane, which was subsequently blocked for 1 h using 5% non-fat dried milk powder. Development of blots was carried out using chemiluminescence in a dark room. Western blots for membrane protein enrichment analysis were probed with the following primary antibodies: anti-caveolin-1 (Ab2910, Abcam) and anti-sodium potassium ATPase (Ab76020, Abcam). Western blot validation of CD55 expression was carried out using anti-CD55 (Ab133684, Abcam). Coomassie blue-stained gels corresponding to the Western blot of CD55 are shown in Supplementary Figure S3.

## 3. Results

### 3.1. Membrane Proteome Coverage

**Figure 1:**
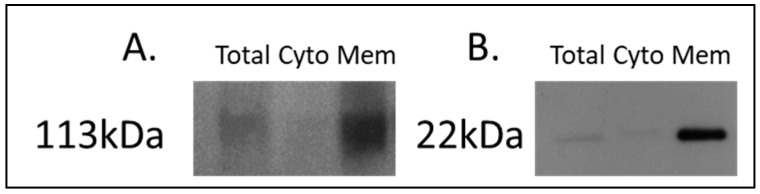
Western blot analysis of (**A**) sodium potassium ATPase and (**B**) caveolin-1 across AsPc1 cell line fractions. A total protein lysate (Total), the cytosolic (Cyto) fraction, and membrane-enriched (Mem) protein fractions from the MemPER kit were analysed. Both membrane protein markers are increased in abundance in the membrane fraction, compared to the total proteome and cytosolic fraction.

For the discovery of candidate human proteins, membrane proteins were enriched from adjacent-normal pancreatic tissues, primary PDAC tumours, and PDAC tumours derived from PDX models at the first (F1) and second (F2) generations, producing a sample set consisting of 40 specimens. The efficiency of the membrane protein enrichment kit was tested using a pancreatic cancer cell line, AsPc1. Figure 1 shows a Western blot of membrane protein-specific enrichment using antibodies against two membrane-specific proteins, caveolin 1 and sodium ATPase. Both Western blots show increased expression for both membrane proteins in the membrane fraction of the kit, an absence in the cytosolic fraction, and low expression in the total protein lysate. These Western blot results confirm that the membrane protein enrichment kit used has successfully selected membrane-associated proteins in the membrane-enriched fraction. 

Quantitative label-free proteomic analysis by LC-MS/MS was performed on the membrane-enriched protein fractions. The total number of protein identifications from enriched fractions for adjacent-normal tissues, PDAC tumours, and PDX F1 and F2 generations, are 2387, 2485, 2617, and 2588, respectively (Supplementary Table S2). Gene Ontology annotation, using the ProteinCenter software within Proteome Discoverer, indicated that membrane-associated proteins comprised 48.8%, 66.5%, 47.8%, and 59.5% of the total proteins identified for adjacent-normal, tumour, PDX F1, and PDX F2 samples, respectively (Supplementary Figure S1).

### 3.2. Differentially Expressed Proteins between Matched Adjacent-Normal and Tumour Tissues

Nine matched adjacent-normal and PDAC tumour tissues were subjected to membrane protein enrichment, and equal concentrations of each fraction were digested with trypsin prior to analysis by mass spectrometry for a “bottom-up” proteomic approach. Raw MS data was interrogated using Progenesis QI for Proteomics label-free LC-MS software, to identify differentially expressed proteins between the two sample groups. The software selects one of the raw files as the reference run to which all other samples were aligned, allowing relative quantitation of proteins between experimental groups to be calculated. 

**Figure 2:**
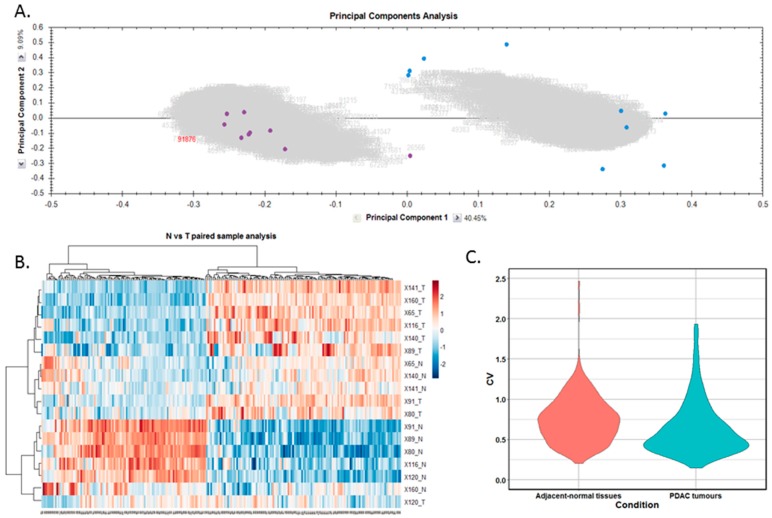
Proteomic clustering of pancreatic cancer tumours and adjacent-normal tissues. (**A**) Principal component analysis from Progenesis QI for Proteomics shows separation of the pancreatic ductal adenocarcinoma (PDAC) tumours (purple, *n* = 9) and the adjacent-normal tissues (blue, *n* = 9). (**B**) Heatmap using unsupervised Pearson clustering of all differentially expressed proteins visualises their expression levels for each sample. (**C**) The CV was calculated for each DE protein in the sample groups, and those values are displayed as violin plots.

Differentially expressed proteins between the adjacent-normal and tumour tissues were determined by using a repeated measures ANOVA *p*-value of <0.05, a fold-change cut-off of ±1.5-fold, and a minimum of 2 unique peptides contributing to a protein identification. Principal component analysis (Figure 2A) and unsupervised Pearson clustering (Figure 2B) of the differentially expressed proteins shows an evident separation of the experimental groups into two categories relating to the adjacent-normal and tumour tissues. Next, we assessed the CV among the patients for each of the sample cohorts, and plotted the distribution as violin plots (Figure 2C). The average CV of the tumour specimens is lower than that of the adjacent-normal group, perhaps due to the heterogeneity of the latter samples. All PDAC tumours were removed from the pancreas, and are all malignant in nature. Adjacent-normal tissues are taken from any region of the pancreas once it is sufficiently distant from the tumour to be deemed non-cancerous, thus leading to a variety of tissue regions acting as an adjacent-normal control and contributing to the degree of variability shown here. The inherent variability across ten patients also adds to the variability seen within both sample groups. 

**Figure 3:**
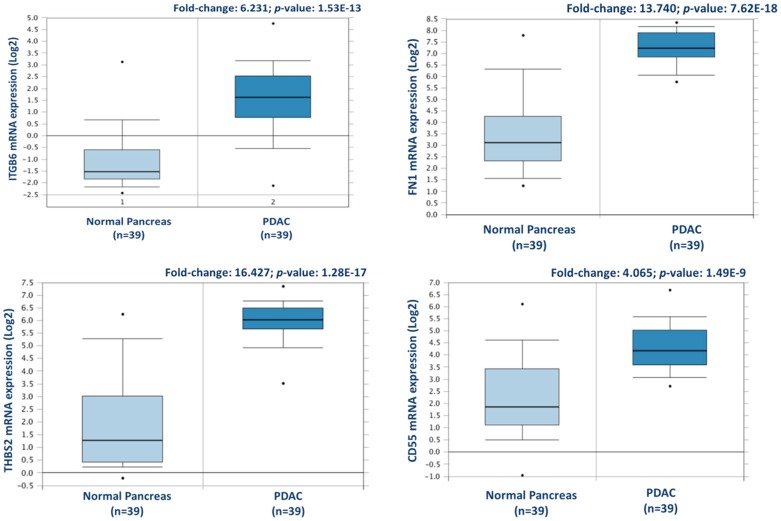
Validation of differential expression levels for 4 candidate proteins across a larger, separate PDAC cohort using Oncomine. *ITGB6*, *FN1*, *THBS2*, and *CD55* gene expression levels were all significantly increased in PDAC (*n* = 39), compared to normal pancreas in the Badea et al. 2008 gene expression set [23].

From this analysis, a total of 238 proteins were identified as differentially expressed between matched adjacent-normal and tumour tissues using paired sample statistical analysis. Within these proteins, 129 proteins were overexpressed and 109 underexpressed in the PDAC tissue. The accession number, number of unique peptides identified, fold-change, and *p*-value of these proteins are outlined in Supplementary Table S3. The top 25 differentially expressed proteins, with increased expression in PDAC tumour tissues compared to adjacent-normal tissues, are shown in Table 1. Of the 129 proteins significantly overexpressed in the PDAC specimens, proteins with the most elevated expression levels, with values of 5-fold or higher, were integrin beta-6, fibronectin, thrombospondin-1 and 2, periostin, immunoglobulin superfamily containing leucine-rich repeat protein, 14-3-3 protein sigma, transforming growth factor beta-1-induced transcript 1 protein, adipocyte enhancer-binding protein 1, and complement decay-accelerating factor (CD55). To further investigate the potential clinical relevance of these identified candidates, we analysed the Badea 2008 gene expression dataset [23] using the Oncomine Research Edition software (Thermo Fisher Scientific), which compares human PDAC cases to normal pancreas tissues. We analysed 4 proteins of interest; ITGB6, FN1, THBS2, and CD55 from our data. The mRNA levels of these candidate proteins are significantly upregulated in the Badea 2008 PDAC tumour dataset (*n* = 39), validating our results in the context of a larger, independent PDAC cohort. This emphasises the potential of these proteins as markers of PDAC for drug targeting or diagnostics (see Figure 3). 

### 3.3. Differentially Expressed Proteins between Matched Tumour and PDX F1 Tissues

#### 3.3.1. Proteome Analysis of PDAC Xenograft Tumours

From the 20 PDAC PDX samples, a total of 2617 and 2588 proteins were identified for the F1 and F2 generation, respectively. These proteins represent a combination of human and mouse identifications, because of the mouse proteome integration with the tumour during the engraftment process [21]. To overcome the challenge of identifying tumour-derived proteins and, thus, potential biomarkers and targets for PDAC, all species-indistinguishable proteins from this study were removed and analysed separately. On average, the protein-coding regions of the mouse and human genomes are 85 percent identical; some genes are 99 percent identical, while others are only 60 percent identical (National Human Genome Research Institute, 2010). This orthologous nature of mouse and human genomes makes it difficult to confidently assign protein identifications from PDX models to one species. The combined human/mouse FASTA database allows the Proteome Discoverer identification software and search engine algorithms to impartially search all MS/MS spectra against every annotated human and/or mouse protein sequence, and provide it with an algorithm score and accession number. Where an MS/MS spectrum matches both the human and mouse counterparts of a protein with equivalence, the corresponding human and mouse accession numbers are reported in the peptide-spectrum match (PSM) field. By filtering for only PSMs which have a single protein match, we can assume that such peptides are species-specific. Such peptide identifications can confidently decipher the origin of the protein, i.e., if it is tumour-derived or mouse-derived. On average, two-thirds of all PSMs from the PDX samples are homologous to the human and mouse counterparts, making them species indistinguishable. The species-specific and species-indistinguishable sets of PSMs were analysed, and differential protein lists were generated. This evolutionary change of amino acids within a protein is advantageous, as it indicates that the identified proteins are most likely derived from the patient PDAC tumour cells proliferating within the mouse tumour microenvironment [21]. Two representative chromatograms of human-specific peptides, identified in this study, and their corresponding BLASTP search results, are shown in Supplementary Figure S2. These results demonstrate the feasibility of identifying large numbers of human-specific proteins from xenograft mouse models of PDAC, and applying this method to other solid tumour xenografts. 

#### 3.3.2. Differential Expression of Human-Specific Proteins

An analysis of the PDAC patient tumours and PDX F1 tumours was carried out to identify candidate proteins and confirm the maintenance of the proteomic profile for PDAC in PDX models. Within this comparison, 9 of the 10 PDX F1 samples were patient-matched to the original tumour specimens (as shown in Supplementary Table S1), allowing a stringent and well-controlled comparison of the PDX and patient tumour proteomes. As described above, differentially expressed proteins between the primary and PDX F1 tumours were determined using an ANOVA *p*-value of <0.05, a fold-change cut-off of ±1.5-fold, and a minimum of 2 unique peptides contributing to a protein identification. For this analysis, only PSMs that matched to a single protein identification and that had an identification as human, and not mouse, were used.

**Figure 4:**
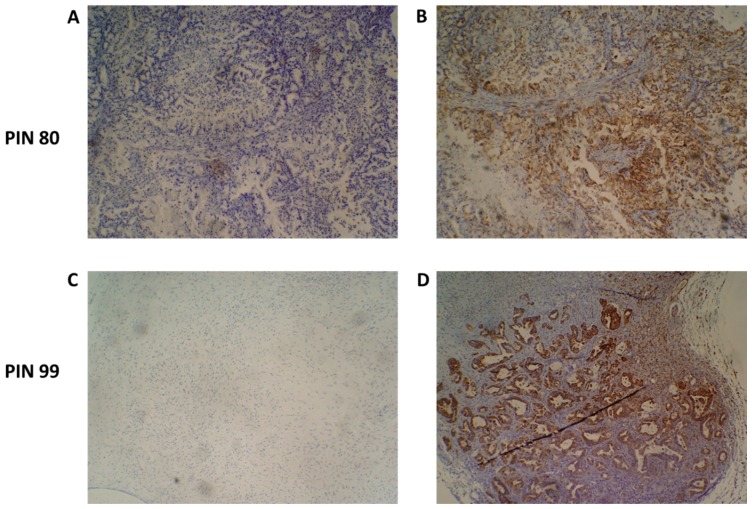
Species-specific immunohistochemical analysis of patient-derived xenograft (PDX) tumour samples. An antibody specific for human mitochondria was used to identify the presence of human cancer cells present in PDX tumours. Representative images of PIN 80 (**A**,**B**) and PIN 99 (**C**,**D**) show negative staining (**A**,**C**) and human species-specific staining of mitochondria (**B**,**D**). Antibody-positive cells are marked by dense brown staining in panels (**B**,**D**).

**Figure 5:**
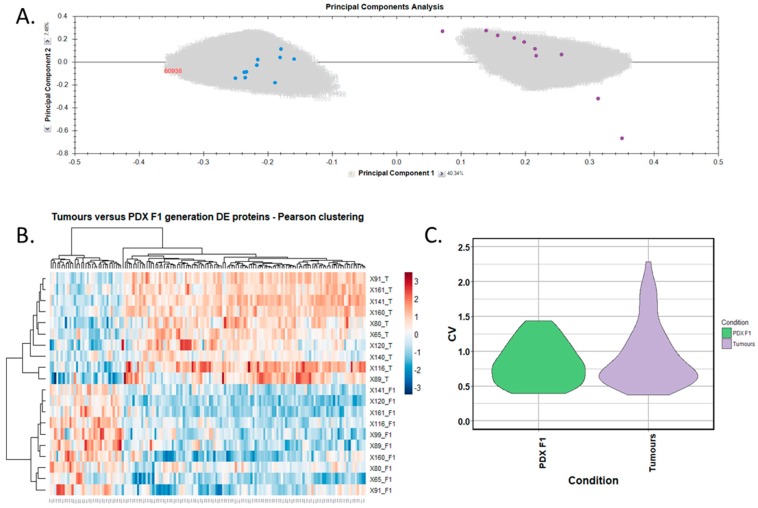
Proteomic clustering of pancreatic cancer tumours and PDX F1 tissues. (**A**) Principal component analysis from Progenesis QI for Proteomics shows a clear separation of PDX F1 (blue, *n* = 10) and the primary PDAC tissues (purple, *n* = 10). (**B**) Heatmap using unsupervised Pearson clustering of all human-specific differentially expressed proteins visualises their expression levels for each sample. (**C**) The CV was calculated for each differentially expressed protein in the sample groups, and those values are displayed as violin plots.

Upon tumour engraftment into the mice, mouse-derived cells replace the non-cancer human cells of the original tumour and, thus, only the cancer cells of the patient tumour remain [24]. Figure 4 shows representative immunohistochemistry staining of two PDX F1 tissue sections for patients 80 and 99, with a human mitochondria-specific antibody. IHC staining in Figure 5B confirms that the stromal component of the PDAC tissues are unstained and, thus, are of mouse origin after engraftment. 

We selected human-only proteins from the differential proteome analysis of PDX F1 versus primary tumours, excluding mouse proteins, as our interest is to identify tumour-specific proteins. A total of 143 differentially expressed, human-specific proteins were identified, including 32 overexpressed and 111 underexpressed in the PDX F1 tumours when compared to the original patient PDAC tumours. The accession number, gene name, number of peptides for identification, and *p*-value for these proteins are outlined in Supplementary Table S3. Principal component analysis (Figure 5A) and unsupervised Pearson clustering (Figure 5B) of the differentially expressed proteins shows an evident separation of the experimental groups into two clear categories corresponding to the tumour tissues and PDX F1 tissues. We determined the CV within the 10-sample set for each differentially expressed protein, and plotted the CV distribution as violin plots (Figure 5C). The average CV of the PDX F1 tumours is lower than that of the patient tumours. This could be a result of the tumour implantation into the mice acting as a selection process whereby only aggressive, highly expressed pivotal proteins are retained in the PDX model, thus yielding a unique signature of proteins with little variation across the multiple specimens. Alternatively, a fraction of the differentially expressed proteins in the patient sample were stroma-derived, and were replaced by mouse proteins in the PDX F1 tumour. The original PDAC patient tumours show greater variation in expression of the differentially expressed proteins, perhaps reflecting interpatient heterogeneity and the different PDAC stages of the resected cancer. The PDX models are a much more controlled study group with regards to, for example, tumour volume and duration of tumour growth. 

These human-specific proteins are potentially compelling biomarkers and targets for pancreatic cancer therapy, as they are tumour cell-associated. Through the PDX engraftment process, the tumours undergo selection pressure, whereby proteins linked to proliferation and responsible for driving growth may increase in expression. The cohort of differentially expressed human proteins with higher abundance levels in the F1 PDX generation thus potentially represent key regulators of PDAC disease progression.

The 32 human proteins with significantly increased expression in the PDX F1 tumour tissues, compared to the patient tumours, are outlined in Table 2. Of these, the most elevated expression levels, with values of 3-fold or higher, were established for periplakin, serpin B5, hydroxymethylglutaryl-CoA synthase, chloride intracellular channel protein 3, integrin beta-4, protein S100-A16, annexin A13, protein S100-A14, and aldo-keto reductase family 1-member B10. 

**Figure 6:**
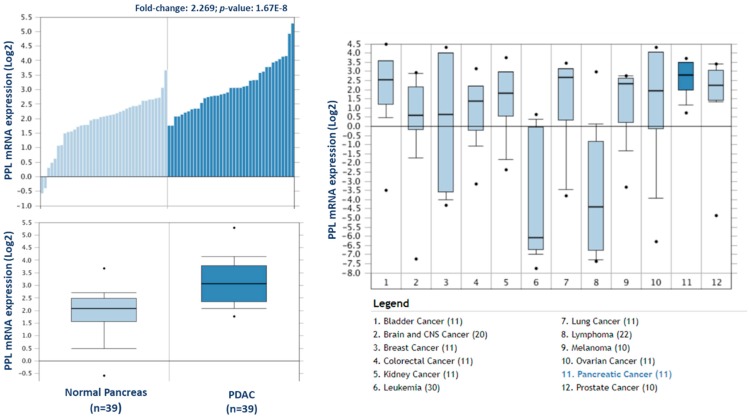
Expression of periplakin in a larger PDAC cohort and across multiple cancer types. mRNA expression of periplakin (*PPL*) is significantly higher in PDAC tumours (*n* = 39) than in normal pancreas (*n* = 39). Similarly, *PPL* mRNA expression is significantly highest in pancreatic cancer compared to 11 other cancer types. The Oncomine database (www.oncomine.org) was used to analyse our candidates’ expression in the Badea 2008 PDAC, and Ramaswamy multi-cancer microarray expression datasets.

To investigate the potential clinical relevance of the highest differentially expressed proteins in F1, we analysed the Badea 2008 microarray gene expression dataset, and the Ramaswamy 2001 multiclass cancer microarray gene expression set using Oncomine software (Figure 6) [23,25]. Periplakin (PPL), which is over 7-fold higher in our cohort of F1 tumours compared to primary tumours, is also significantly overexpressed in the Badea gene expression set of PDAC tumours (*n* = 39) compared to normal pancreas tissue (*n* = 39). *PPL* also showed the highest gene expression in PDAC compared to 11 other cancer types in the Ramaswamy 2001 multi-cancer gene expression set. This result illustrates that our proteomic data validates in a larger, separate PDAC cohort, and that targets identified here have strong potential as therapeutic targets for PDAC. 

#### 3.3.3. Species-Indistinguishable Proteins

The human-specific proteins identified above have high potential as PDAC tumour biomarkers. However, two-thirds of the significantly differential PSMs between PDX F1 and primary PDAC tumours could not be assigned definitively to a single species, because the MS/MS analysis and search software did not detect species-unique peptide(s) which could determine whether those proteins are derived from mouse (stromal) cells or human (cancer) cells. A total of 599 differential proteins were identified by peptides common to both human and mouse sequences and are, therefore, species-indistinguishable identifications. Of these, 330 proteins were overexpressed, and 269 were underexpressed in the PDX F1 tissues, compared to the original PDAC tumours. The accession number, fold-change, number of peptides, and *p*-value of these proteins, are outlined in Supplementary Table S3.

Identifications using a dual database for xenograft studies for which PSMs report two matching proteins that are not species-unique may be false-positive identifications. For example, of the 269 proteins with higher abundance in the PDAC primary tumours compared to the mouse PDX tumours, 206 are assigned to the human species whereas 63 are identified as mouse. The latter group represents an impossibility, as the original patient tumours contain no mouse proteins, and these are false-positive identifications that should have been assigned as human. These candidate markers are classified as species-indistinguishable proteins. Since this large significantly differential indistinguishable cohort potentially presents an interesting set of proteins that may play a role in tumour progression, they deserve closer inspection.

There were 330 indistinguishable proteins identified with a higher abundance in PDX F1, compared to primary PDAC tumours. This cohort of differential proteins represents a persistent aggressive subset of proteins which have the capabilities to survive tumour engraftment and proliferate. Some of the most differentially expressed proteins, with a fold-change of 10 or more, include platelet glycoprotein 4, probable ATP-dependent RNA helicase DDX17, xanthine dehydrogenase/oxidase, tropomyosin alpha-4 chain, von Willebrand factor A domain-containing protein 5A, thymosin beta-4, serum albumin, granulins, annexin A11, NADH dehydrogenase [ubiquinone] 1 alpha subcomplex subunit 8, and lysosomal protective protein. 

### 3.4. Stability of Protein Expression over PDX Generations

The stability of PDX models of pancreatic cancer for biomarker discovery was evaluated by profiling the tumour proteome over various PDX generations. Pancreatic tumours harvested from the first generation in mice were expanded into further generations. Tumour fragments were stored at each generation, and subjected to label-free proteomic analysis. Supplementary Table S1 illustrates the success of the pancreatic PDX models generated in-house with most PDX F1 tumours progressing to successful engraftment and expansion in the F2 PDX generation of mice. Ten F1 generation and ten F2 generation PDX tumours were analysed, with 9 out of 10 of these tumours being patient-matched. Quantitative label-free differential analysis of the F1 and F2 tumours identified a small number of differentially expressed proteins, with 8 human-specific proteins (Table 3), and 41 proteins of indistinguishable species (Supplementary Table S3) as being significantly differential in abundance levels. 

The 8 altered human-specific proteins include 5 proteins with an increased expression in the F2 generation compared to the F1 PDX generation. These proteins potentially signify an aggressive cohort of proteins whose expression increased over two stages of engraftment. From this cohort, 4 out of 5 of the proteins (IDH1, CTSD, PRDX6, and SERPINB6) also showed a statistically significant increase in abundance (12, 2.3, 2.6, and 3-fold, respectively) in the patient tumour samples when compared to the PDX F1 generation in the species-indistinguishable study. Although their expression is not significantly increased in the F1 generation, they are still present, and it is not until the second engraftment (F2) that their expression levels increase. 

Species-indistinguishable proteins were also profiled in the F1 versus F2 PDX generations, to investigate proteomic changes over the PDX generations. In this analysis, 41 proteins were identified as significantly differentially expressed, with 22 proteins having an increased expression in F2, and 19 having a decreased expression in F2 compared to F1. 

This analysis demonstrates the stability of the human tumour proteome in the PDX mice models of pancreatic cancer. Of the human-specific proteins, only 5 show a significant change in their abundance levels within the F2 generation and, of those, none surpass a 2-fold difference, indicating the change is minimal, and unlikely to be phenotypic. This analysis is the first, to our knowledge, that profiles the human-specific proteomic changes of patient-derived tumours from the point of surgical resection through to the different PDX generations. 

### 3.5. Western Blot Analysis of CD55

**Figure 7:**
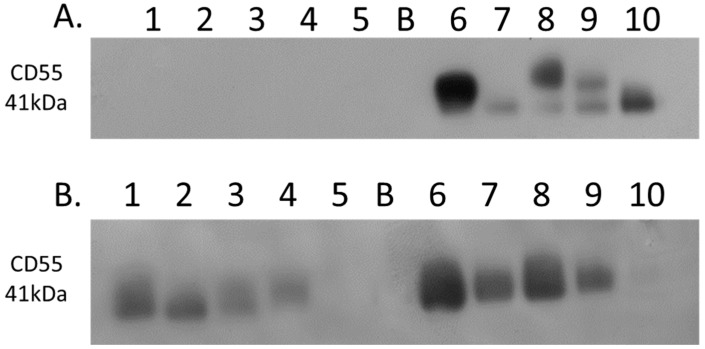
Western blot analysis of CD55 expression. (**A**) Expression of CD55 across 5 representative samples from adjacent-normal and primary PDAC tumours (Lanes 1–5: 89N, 91N, 116N, 120N, and 161N; Lane B: Blank; Lanes 6–10: 65T, 120T, 140T, 160T, and 161T). CD55 is absent from the adjacent-normal tissue set and present in all 5 of the primary PDAC membrane-enriched fractions. (**B**) Expression of CD55 across 5 representative membrane-enriched fractions from PDX F1 and F2 tumours (Lane 1–5: 89F1, 116F1, 141F1, 160F, and 161F1; Lane B: Blank; Lanes 6–10: 65F2, 80F2, 89F2, 99F2, and 160F2). CD55 is expressed in 4 out of 5 tumours for both F1 and F2 sets.

Complement decay-accelerating factor (CD55) was identified as significantly differentially expressed between primary PDAC tumours and matched adjacent-normal tissues, with a 6.77-fold increase in expression in PDAC tissue. Western blot analysis was used to verify the presence of CD55 across representative samples from adjacent-normal, primary PDAC, PDX F1, and PDX F2 tumours (Figure 7). CD55 is expressed in all 5 of the primary PDAC tumours, and in 4/5 of the PDX F1 and F2 tumour tissues. It is absent in all 5 adjacent-normal tissue analysed, suggesting it has strong specificity for PDAC. Coomassie-stained gels to show loading are shown in Supplementary Figure S3. When analysed across the Badea microarray gene expression set (Figure 3), *CD55* was found to have a 4.065-fold increase in the PDAC tumours (*n* = 39) compared to normal pancreas. These results, taken together, provide evidence that CD55 has potential as a target for PDAC.

## 4. Discussion

Pancreatic cancer has traditionally been characterised in the clinic through expression of CA19-9 in the blood, despite the median sensitivity of CA19-9 for diagnosis being 79% and median specificity 82% [26]. In addition, several factors influence CA19-9 levels, including the presence of jaundice, which elevates CA19-9, leading to false-positives, and also masking disease progression. The lack of sensitivity and specificity shown by CA19-9 demonstrates the need for novel diagnostic or prognostic biomarkers for PDAC. 

The development of effective chemotherapies also remains a challenge in PDAC. Gemcitabine is the standard chemotherapy for PDAC and many other cancer types; its incorporation into the DNA leads to inhibition of DNA synthesis and, ultimately, cell death. Although it has been the first-line treatment in patients of PDAC, its use alone achieves only modest survival benefits, with the 1-year survival rate being 18% in those treated with gemcitabine [27]. As most approved drugs target proteins, the evaluation of pancreatic cancer proteomes holds promise for identifying PDAC-specific drug targets. The proteomic profiling of PDAC tumours, in this study, has established discrete changes in the abundance and expression pattern of many proteins resulting from the disease process. Differential expression of such targets potentially could be manipulated using novel drug schemes, thus improving the treatment of pancreatic cancer, and/or could be employed as novel biomarkers for diagnosis or monitoring disease progression and the efficacy of treatment. Here, we have analysed the proteomic profiles of 9 adjacent-normal patient-matched control pancreatic specimens, 10 PDAC tumours, and 20 matched pancreatic cancer patient-derived xenograft tumours. The quantitative label-free LC-MS-based proteomic analysis of membrane protein changes in these samples identified protein targets of PDAC which could potentially be used in a targeted therapeutic drug scheme, such as ADC therapy. The sample preparation method used enriches for membrane proteins, but is not an absolute fractionation, so that proteins from other cellular compartments, in particular, abundant cytoplasmic proteins, such as enzymes, also appear in our lists. Although these proteins are not of interest as candidates for membrane-targeted therapeutics, such as ADCs, some of the cytosolic proteins identified warrant future analyses to determine their role in PDAC development. Importantly, this study has established that PDX tumours recapitulate the proteomic signatures of human pancreatic cancers. This profile is narrowly altered in the subsequent F2 generation in PDX mice, which highlights their value as both therapeutic targets and for generating larger masses of tumour material for study than is typically available from the primary tumour sample. 

From our analysis of the patient-matched adjacent-normal and pancreatic tumours, 238 proteins were identified as significantly differentially expressed with a minimum fold change of 1.5-fold between the experimental groups. Over 10 of these proteins exhibit a substantial increase in abundance levels with greater than 5-fold level of overexpression in tumours compared to the adjacent-normal group. Of these, integrin beta-6, a member of the integrin family, is well-studied for its function of attaching the cell cytoskeleton to the extracellular matrix, thus explaining its presence in the membrane-enriched fraction of the pancreatic adenocarcinomas [28,29]. Integrin beta-6 specifically functions as a receptor for fibronectin, which justifies the high levels of this protein that function at the cell membrane in adhesion and motility [30]. The identification and role of this integrin receptor as a fibronectin-binding protein was first characterised in a pancreatic carcinoma cell line FG-2 in 1992, highlighting its importance in this cancer [30]. Thrombospondin 1 and 2, also overexpressed in PDAC tumours, are adhesive glycoproteins that mediate cell-to-cell and cell-to-matrix interactions [31]. Complement decay-accelerating factor (CD55) has been identified by immunohistochemistry in pancreatic cancer, and its elevated levels correlate positively with tumour aggressiveness, vascular invasion, and prognosis [32]. The increased levels of CD55 in pancreatic adenocarcinoma specimens suggest a protective role to prevent tumour cells from bystander killing by complement [33]. We found a 5.58-fold increase in abundance of CD55 in the PDAC samples compared to the normal-adjacent samples from the proteomic analysis. This increased expression in PDAC patients was validated by Western blotting across adjacent-normal and tumour samples, and was also confirmed to maintain increased expression in the PDX models. TGF beta-1-induced transcript 1 protein (Hic-5) has not been reported to play a role in pancreatic cancer, to date, however, it has been long-studied in prostate cancer, and its expression is suggested to correlate with tumorigenesis [34,35]. More recently, the expression of Hic-5 has been shown to be involved in the modulation of the stromal matrix in prostate and breast cancer, as well as other models of the stromal matrix [36,37]. These proteins, which exhibit a strongly significant and considerably increased abundance specific to the cohort of PDAC tumours, have potential as both novel drug targets and biomarkers for PDAC. In an attempt to validate our findings over a larger separate cohort of PDAC data, we used the bioinformatic tool Oncomine, which houses microarray gene datasets from various studies worldwide. Four differentially expressed proteins were analysed against the Badea 2008 PDAC gene microarray dataset, which contains differential expression information for 400 genes across normal pancreas (*n* = 39) and PDAC (*n* = 39). In that dataset, all four upregulated proteins (ITGB6, FN1, THBS2, and CD55) were similarly found to be overexpressed in PDAC, compared to normal pancreas. The retrieval of these potential markers, which have been found previously by microarray analysis of a larger PDAC cohort, shows the value of quantitative proteomic expression analysis to identify markers for pancreatic cancer that represent candidate proteins for drug targets of PDAC disease. 

Proteomic analysis of the PDX tumours we generated proved challenging in terms of identifying the protein targets that are undeniably derived from the original patient PDAC tumour. By using human-specific peptide sequences to identify patient tumour marker proteins, we were able to identify 32 significantly differentially expressed proteins having an increased abundance in the PDX F1 tumours compared to primary PDAC tumours. These proteins represent an exceptionally unique cohort of the PDAC proteome that has survived engraftment and, also, adapted successfully to the murine tumour microenvironment with higher expression levels than in the original tumour. Most interestingly, periplakin (PPL) had the highest change in expression levels, with a 7.85-fold increase in PDX F1 compared to primary PDAC tumours. PPL is a component of desmosomes, which are present on the cell membrane and allow for cell–cell attachment. Although periplakin has been previously identified in pancreatic cancer, it has never been the focus of study [38]. PPL has been extensively studied as a binding partner of Akt [39], which is well-established for its role in cell proliferation, migration, apoptosis, and various cancers. This may indicate a role for PPL in PDAC tumour progression through Akt, and its increased expression in the PDX F1 tumour cohort suggests it may be driving tumour growth by increasing proliferation. This significantly increased expression in the PDX F1 tumours suggests an important role for this protein in PDAC tumorigenesis, which warrants future investigation.

This is the first study to profile the PDAC PDX tumour proteome through 2 generations after initial engraftment. This analysis identified 8 human-specific proteins and 41 species-indistinguishable proteins with significant differential expression. Of those 49 altered proteins, 30 were overexpressed in the F2 tumours compared to the F1 cohort. This analysis demonstrates that the PDAC PDX model used here largely retains the primary patients’ PDAC proteomic profile over time and various generations, and provides great encouragement for applying PDX models for successful PDAC research.

The comparison of adjacent-normal tissues to primary PDAC tumours has the advantage that all proteins identified are human, but the limitation is that the tumours contain variable amounts of admixed normal cells (stromal cells, immune system cells, etc.) and this tends to dampen the discriminatory power of the differential expression analysis. The comparison of primary tumours vs. xenograft tumours, when confined to “human-specific”, may be the richer list for candidate target identification. These differentially expressed proteins are from a comparison of the primary tumour biopsy sample and the pure tumour cells as, during the xenograft process, the human stromal cells are lost and replaced by mouse stroma, and the latter does not contribute to the human-specific protein list. We, the authors, believe these proteins could be more reliable PDAC targets for future investigations. 

## 5. Conclusions

Our investigation has identified proteins that are significantly differentially expressed in primary PDAC tumours compared to patient-matched adjacent-normal tissues. We present 129 proteins that are overexpressed in PDAC that have potential as therapeutic targets or biomarkers of disease. Importantly, we validated the specificity of 4 differentially expressed proteins across a microarray gene expression set from an independent cohort of PDAC patients. For the first time, proteomic profiling of PDAC PDX models, as described in this study, demonstrates that PDX tumours recapitulate the primary patient’s PDAC tumour over time and various generations. Through IHC, we established that much of the stromal component of the tumours is overcome by mouse cells once engrafted, and only human tumour cells are retained. This occurrence is advantageous, and allowed us to identify tumour cell-associated proteins in PDAC upon PDX engraftment. These proteins represent strong candidates for novel therapeutic targeting, such as ADC therapy. Of course, the xenografts are not perfect models of human pancreatic cancer because they differ in anatomical location, host species differences, and the presence of murine stroma, but they nevertheless provide a rich source of human-derived pancreatic cancer cells, in situ, in a stromal environment, for molecular studies. This study is the first comparative proteomic analysis of PDAC that employs PDX models to identify patient tumour cell-associated proteins to identify potential targets for novel therapeutic treatment of PDAC.

## Figures and Tables

**Table 1:** List of top 25 differentially expressed proteins with increased expression in PDAC tumour tissues compared to adjacent-normal tissues as determined by quantitative label-free mass spectrometric analysis using repeated measures ANOVA for paired sample analysis.

Accession	Protein Name	Gene	Peptides	*p*-Value	Fold-Change
P18564	Integrin beta-6	*ITGB6*	2	8.80 × 10^−3^	8.69
P02751	Fibronectin	*FN1*	3	4.67 × 10^−2^	7.95
P07996	Thrombospondin-1	*THBS1*	5	2.56 × 10^−2^	7.65
Q15063	Periostin	*POSTN*	8	2.06 × 10^−2^	7.34
O14498	Immunoglobulin superfamily containing leucine-rich repeat protein	*ISLR*	2	4.27 × 10^−2^	7.10
P31947	14-3-3 protein sigma	*SFN*	3	1.19 × 10^−3^	6.06
P35442	Thrombospondin-2	*THBS2*	5	1.33 × 10^−2^	6.02
O43294	Transforming growth factor beta-1-induced transcript 1 protein	*TGFB1I1*	2	5.98 × 10^−3^	5.75
Q8IU×7	Adipocyte enhancer-binding protein 1	*AEBP1*	3	1.72 × 10^−2^	5.59
P08174	Complement decay-accelerating factor	*CD55*	2	8.43 × 10^−4^	5.58
Q03518	Antigen peptide transporter 1	*TAP1*	2	4.62 × 10^−3^	5.00
Q03519	Antigen peptide transporter 2	*TAP2*	2	2.42 × 10^−2^	4.24
O15533	Tapasin	*TAPBP*	2	1.14 × 10^−2^	4.07
P23142	Fibulin-1	*FBLN1*	6	1.44 × 10^−2^	3.81
Q08380	Galectin-3-binding protein	*LGALS3BP*	3	7.24 × 10^−3^	3.80
P50454	Serpin H1	*SERPINH1*	2	5.73 × 10^−4^	3.64
P02792	Ferritin light chain	*FTL*	3	3.77 × 10^−2^	3.56
P98095	Fibulin-2	*FBLN2*	2	1.10 × 10^−2^	3.41
P20592	Interferon-induced GTP-binding protein Mx2	*MX2*	2	2.43 × 10^−4^	3.38
Q6UX06	Olfactomedin-4	*OLFM4*	3	4.26 × 10^−2^	3.27
Q01518	Adenylyl cyclase-associated protein 1	*CAP1*	2	3.22 × 10^−2^	3.22
Q01995	Transgelin	*TAGLN*	6	2.51 × 10^−2^	3.20
Q96HE7	ERO1-like protein alpha	*ERO1A*	11	2.39 × 10^−3^	3.16
Q92597	Protein NDRG1	*NDRG1*	2	3.28 × 10^−2^	3.15
Q6PIU2	Neutral cholesterol ester hydrolase 1	*NCEH1*	4	1.51 × 10^−5^	3.13

**Table 2:** The 32 human-specific proteins identified with significantly increased expression in PDX F1 tumours compared to PDAC tumour tissues, as determined by quantitative label-free mass spectrometric analysis.

Accession	Protein Name	Gene	Peptides	ANOVA (*p*)	Fold Change
O60437	Periplakin	*PPL*	2	3.34 × 10^−8^	7.85
P36952	Serpin B5	*SERPINB5*	7	2.37 × 10^−3^	4.86
P54868	Hydroxymethylglutaryl-CoA synthase, mitochondrial	*HMGCS2*	3	4.29 × 10^−3^	4.79
O95833	Chloride intracellular channel protein 3	*CLIC3*	2	6.99 × 10^−3^	3.62
P16144	Integrin beta-4	*ITGB4*	2	6.03 × 10^−4^	3.35
Q96FQ6	Protein S100-A16	*S100A16*	3	9.17 × 10^−4^	3.28
P27216	Annexin A13	*ANXA13*	6	2.72 × 10^−3^	3.27
Q9HCY8	Protein S100-A14	*S100A14*	4	5.20 × 10^−3^	3.09
O60218	Aldo-keto reductase family 1-member B10	*AKR1B10*	3	2.04 × 10^−2^	3.03
P11047	Laminin subunit gamma-1	*LAMC1*	2	9.87 × 10^−3^	2.97
Q12864	Cadherin-17	*CDH17*	4	2.27 × 10^−2^	2.81
P09327	Villin-1	*VIL1*	3	5.26 × 10^−4^	2.68
Q9H190	Syntenin-2	*SDCBP2*	2	1.15 × 10^−3^	2.67
P05787	Keratin, type II cytoskeletal 8	*KRT8*	5	1.51 × 10^−2^	2.63
P15311	Ezrin	*EZR*	2	2.79 × 10^−3^	2.60
P56470	Galectin-4	*LGALS4*	7	1.09 × 10^−2^	2.58
Q14764	Major vault protein	*MVP*	5	2.31 × 10^−3^	2.42
Q8NFV4	Alpha/beta hydrolase domain-containing protein 11	*ABHD11*	4	6.45 × 10^−3^	2.40
P42765	3-ketoacyl-CoA thiolase, mitochondrial	*ACAA2*	2	7.25 × 10^−4^	2.14
P99999	Cytochrome c	*CYCS*	2	6.30 × 10^−3^	2.14
P08727	Keratin, type I cytoskeletal 19	*KRT19*	9	2.03 × 10^−2^	2.11
P17931	Galectin-3	*LGALS3*	2	1.92 × 10^−2^	2.10
P55011	Solute carrier family 12 member 2	*SLC12A2*	2	2.33 × 10^−2^	2.04
P22307	Non-specific lipid-transfer protein	*SCP2*	2	2.01 × 10^−2^	1.99
P09972	Fructose-bisphosphate aldolase C	*ALDOC*	2	1.83 × 10^−3^	1.94
P12830	Cadherin-1	*CDH1*	3	1.57 × 10^−3^	1.88
Q14126	Desmoglein-2	*DSG2*	4	7.28 × 10^−5^	1.84
Q96I24	Far upstream element-binding protein 3	*FUBP3*	2	1.23 × 10^−2^	1.83
Q02218	2-oxoglutarate dehydrogenase, mitochondrial	*OGDH*	2	1.60 × 10^−3^	1.82
Q9NR45	Sialic acid synthase	*NANS*	3	1.48 × 10^−2^	1.61
P14618	Pyruvate kinase PKM	*PKM*	3	3.90 × 10^−2^	1.56
P55072	Transitional endoplasmic reticulum ATPase	*VCP*	2	1.50 × 10^−2^	1.51

**Table 3:** The 8 significantly differentially expressed human-specific proteins in a comparison of F1 and F2 tumours. The first 5 proteins have an increased expression in the PDX F2 generation compared to F1 tumours, whereas the last 3 proteins listed have higher expression levels in the F1 tumours compared to the F2 tumour group.

Accession	Protein Name	Highest in	Gene	Peptides	ANOVA (*p*)	Fold Change
O75874	Isocitrate dehydrogenase [NADP] cytoplasmic	F2	*IDH1*	2	1.22 × 10^−2^	1.60
P07339	Cathepsin D	F2	*CTSD*	2	2.06 × 10^−2^	1.62
P30041	Peroxiredoxin-6	F2	*PRDX6*	2	2.18 × 10^−2^	1.81
P00352	Retinal dehydrogenase 1	F2	*ALDH1A1*	4	2.36 × 10^−2^	1.76
P35237	Serpin B6	F2	*SERPINB6*	2	2.85 × 10^−2^	1.87
P02768	Serum albumin	F1	*ALB*	3	3.26 × 10^−2^	5.15
P04083	Annexin A1	F1	*ANXA1*	3	3.92 × 10^−2^	2.41
P08133	Annexin A6	F1	*ANXA6*	2	4.19 × 10^−2^	2.58

## Supplementary Materials

The following are available online at http://www.mdpi.com/2227-7382/6/4/45/s1, Figure S1: Cellular component annotation using Gene Ontology analysis through the Protein Center node in Proteome Discoverer for each of the sample sets, Figure S2: MS/MS spectra of two human-specific peptides identified from the PDAC PDX F1 generation, Figure S3: Coomassie stained gels validating loading of lanes prior to the Western blot of CD55, Table S1: Patient demographics, Table S2: Qualitative lists, Table S3: Quantitative lists.
