# Supplementary material for: A Comparative Quantitative LC-MS/MS Profiling Analysis of Human Pancreatic Adenocarcinoma, Adjacent-Normal Tissue, and Patient-Derived Tumour Xenografts
Source: Proteomes. 2018 Nov 6;6(4):45. doi: 10.3390/proteomes6040045 (PMC6313850; doi:10.3390/proteomes6040045)
Supplement: Supplementary file 1 [file proteomes-06-00045-s001.zip › proteomes-381650-supplementary-final/Supplementary Figures.pdf]

*Supplementary Figure S1. Cellular component annotation using Gene Ontology analysis through the ProteinCenter node in Proteome Discoverer for each of the sample sets.*

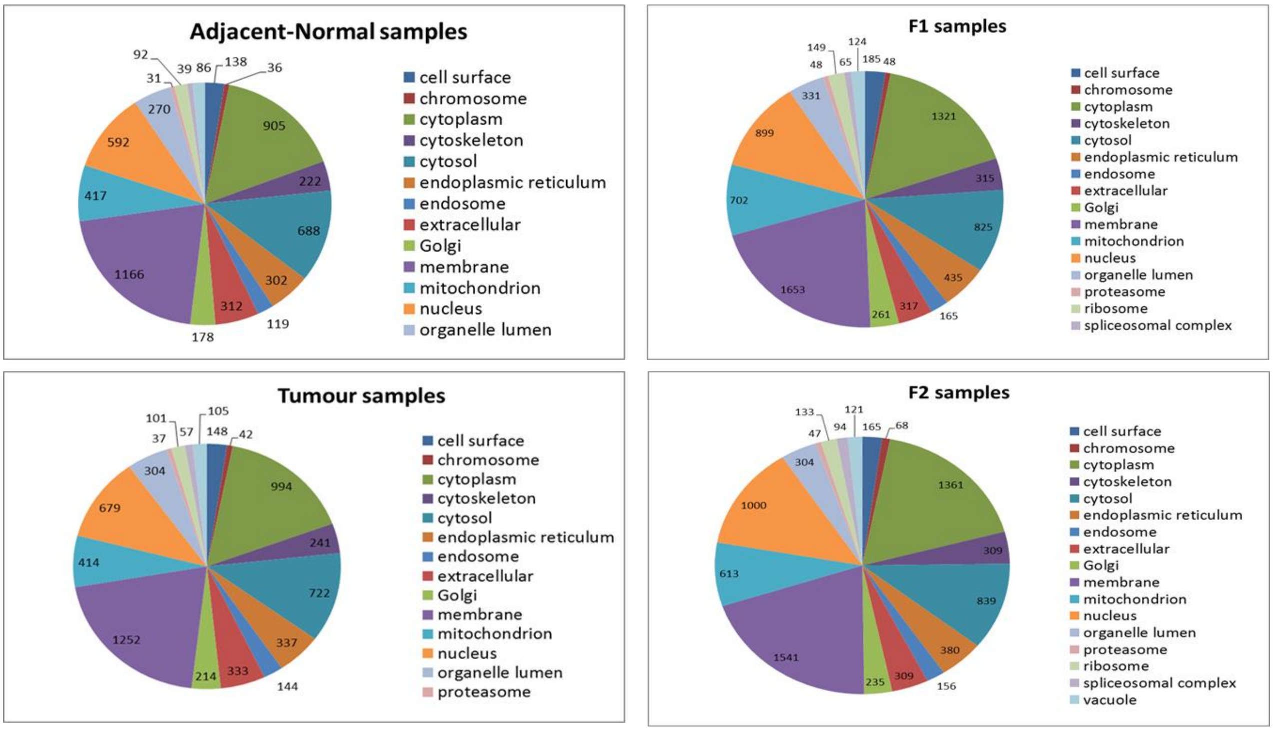


The efficiency of membrane protein enrichment was assessed using gene ontology annotation by the ProteinCenter software. Supplementary figure 1 shows the number of proteins from each sample set in relation to their cellular component annotation. Membrane-associated proteins relate to 48.8%, 66.5%, 47.8% and 59.5% of the total proteins identified for adjacent-normal, tumour, PDX F1 and PDX F2 samples.

**CLIC-3 (77% identity)**

**Human: APLEHELAGEPQL**

**Mouse: APLDHELAQEPHL**

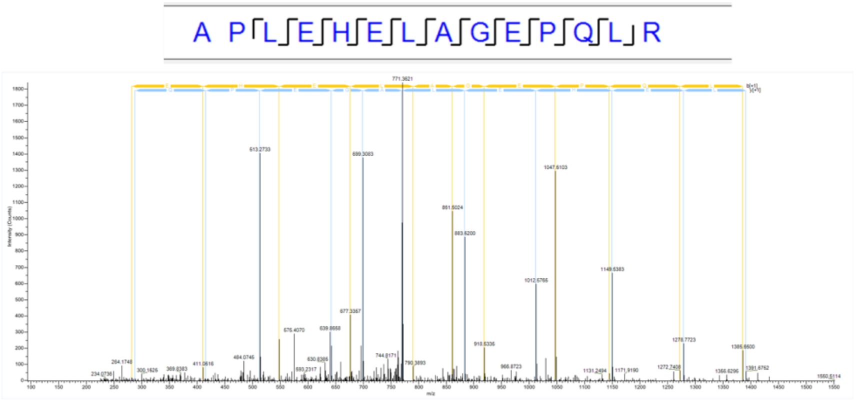


**Protein S100-A14 (80% identity)**

**Human: NFHQYSVEGGKETLTPSELR**

**Mouse: NFHKYSVAGKKETLTPAELR**

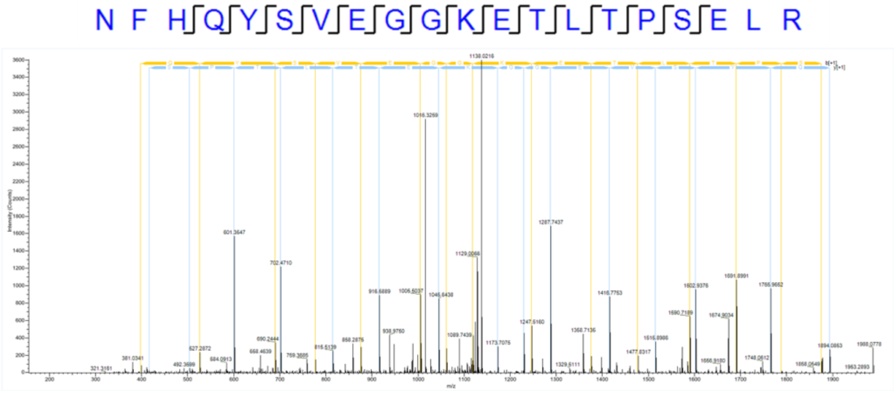


*Supplementary Figure S2: MS/MS spectra of two human-specific peptides identified from the PDAC PDX F1 generation*. Sequence alignments using BLASTp of the identified human peptides to their corresponding mouse peptides are shown. The protein sequence homology between the two species is indicated.

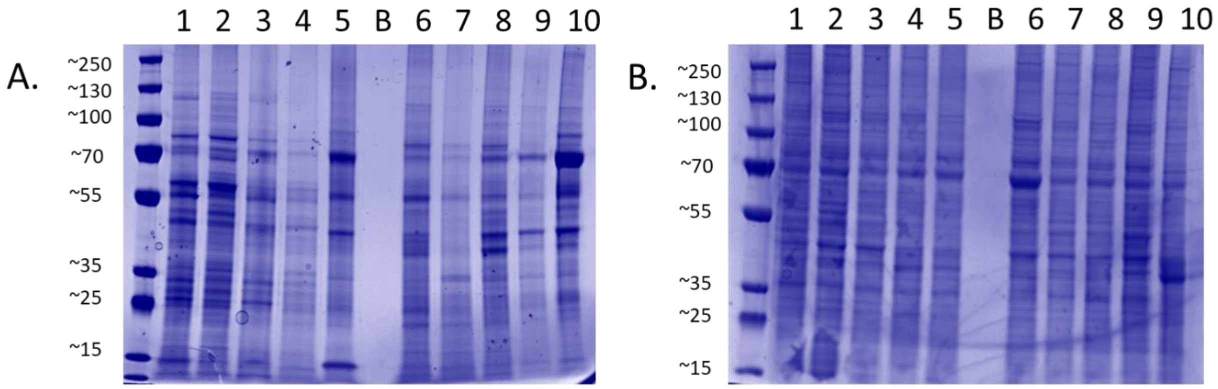


*Supplementary Figure S3: Coomassie stained gels validating loading of lanes prior to the Western blot of CD55*. Coomassie stained gel of (A) Five representative adjacent-normal tissues and primary PDAC tumours membrane protein enriched fractions (Lanes 1-5: 89N, 91N, 116N, 120N and 161N, Lane B is a Blank, Lanes 6-10: 65T, 120T, 140T, 160T and 161T). Coomassie stained gel of (B) 5 representative membrane-enriched fractions from PDX F1 and F2 tumours (Lane 1-5:89F1, 116F1, 141F1, 160F, 161F1, Lane B: Blank, Lanes 6-10: 65F2, 80F2, 89F2, 99F2 and 160F2).
