# Supplementary material for: A Comparative Quantitative LC-MS/MS Profiling Analysis of Human Pancreatic Adenocarcinoma, Adjacent-Normal Tissue, and Patient-Derived Tumour Xenografts
Source: Proteomes. 2018 Nov 6;6(4):45. doi: 10.3390/proteomes6040045 (PMC6313850; doi:10.3390/proteomes6040045)
Supplement: Supplementary file 1 [file proteomes-06-00045-s001.zip › proteomes-381650-supplementary-final/Supplementary Table S1 Patient demographics.pdf]

				Type of tissue specimen analysed			
**Patient I.D.**	**Original Patient**	**Gender**	**Age at surgical resection**	**Adjacent-Normal**	**Tumour**	**F1**	**F2**
PIN 65	Tumour & normal invasive ductal adenocarcinoma	F	76	✓	✓	✓	✓
PIN 80	Tumour & normal invasive ductal adenocarcinoma	M	70	✓	✓	✓	✓
PIN 89	Tumour & normal invasive ductal adenocarcinoma	F	45	✓	✓	✓	✓
PIN 91	Tumour & normal invasive ductal adenocarcinoma	M	64	✓	✓	✓	✓
PIN 99	Tumour & normal invasive ductal adenocarcinoma	F	63	-	-	✓	✓
PIN 116	Tumour & normal invasive ductal carcinoma	M	62	✓	✓	✓	-
PIN 120	Invasive ductal adenoca–HOP (head of Pancreas)	M	65	✓	✓	✓	✓
PIN 140	Tumour & normal invasive ductal carcinoma	M	63	✓	✓	-	✓
PIN 141	Tumour & normal invasive ductal adenocarcinoma	M	82	✓	✓	✓	✓
PIN 160	Tumour & normal invasive ductal adenocarcinoma	F	77	✓	✓	✓	✓
PIN 161	Tumour & normal invasive ductal adenocarcinoma	M	61	✓	✓	✓	✓

Table 1: Characteristics of patients and tissue samples used for LC-MS/MS analysis.
